# De novo identification of the specificities of recurrently identified human T cell receptors

**DOI:** 10.1126/sciadv.aeb1732

**Published:** 2026-02-13

**Authors:** Mithila Kasbe, Berkay Yahsi, Kalyn Whitehead, Jiwon Oh, Mashwiyat Mosharraf, Kayla Sohn, Trinh Phan, Mark N. Lee

**Affiliations:** ^1^ Department of Laboratory Medicine, Yale School of Medicine, 330 Cedar St., New Haven, CT 06519, USA.; ^2^ Human and Translational Immunology Program, Yale School of Medicine, 300 Cedar St., New Haven, CT 06519, USA.; ^3^ Cancer Immunology Program, Yale Cancer Center, 35 Park St., New Haven, CT 06519, USA.; ^4^ Department of Pathology, Yale School of Medicine, 310 Cedar St., New Haven, CT 06519, USA.

## Abstract

T cell repertoires of different individuals occasionally converge on the same T cell receptor (TCR) sequence as a solution to target immunodominant epitopes. A complete mapping of these “public” TCR specificities may enable a global understanding of population-level immune histories. Here, we sought to determine the antigen specificities of public TCRs with unknown target identity. We developed a functional screening workflow in which we screen panels of TCRs for reactivity to individual viral genomes or to ~1000 viral reference strains and then sort out the immunogenic peptides by labeling antigen-presenting cells that are in proximity to activated T cells. Using this workflow, we identified the target specificities of T cells that are circulating in up to 14% of individuals, including a pre–COVID-19 seasonal coronavirus-reactive TCR that cross-reacts with peptides within pandemic coronaviruses; an influenza B–reactive TCR that targets a highly conserved epitope; and TCRs targeting Herpesviridae family viruses that cause long-term latent infections. Our results demonstrate an efficient strategy to reveal public T cell memories de novo, offering a window into shared immune exposures.

## Teaser

A high-throughput screening system enabled identification of the antigen targets of T cell receptors found in many individuals.

## INTRODUCTION

Memories of past viral exposures are preserved in the DNA of T cells within their recombined T cell receptor (TCR) genes and can be read out using TCR sequencing (*1*, *2*). Decoding the target antigen(s) of each TCR sequence enables interpretation of this exposure record. A subset of TCR sequences has been recurrently found within clonally expanded T cells in different individuals and associated with similar past exposure history, e.g., to infection by common human viruses (*1*–*4*). Thus, these “public” TCR sequences are biomarkers of specific viral exposures. A complete mapping of public TCR specificities may enable a global understanding of disease exposures in each individual.

Most public TCR sequences have unknown specificities (*3*). Databases/datasets that have tracked accumulated knowledge of public TCR specificities are dominated by epitopes within cytomegalovirus (CMV), Epstein-Barr virus (EBV), influenza (Flu) A, and the pandemic coronavirus severe acute respiratory syndrome coronavirus 2 (SARS-CoV-2) (*3*, *5*, *6*). Beyond these viruses, public TCRs targeting many other common exposures, including seasonal (“common cold”) human coronaviruses (HCoV) and herpes simplex viruses (HSV), etc., are nearly absent from these databases, despite the high incidence of these infections within the human population (*7*, *8*). A more complete mapping of public TCR specificities will aid in the interpretation of T cell sequencing data (*9*–*12*), as well as in the development of blood-based diagnostics (*1*, *2*, *13*) and antiviral therapeutics (*14*, *15*).

A major bottleneck in the effort to identify public TCR specificities has been the difficulty to test large numbers of putative viral peptides for immunogenicity due to scaling limitations of epitope identification assays that are in widespread use, including tetramers and ELISPOT (enzyme-linked immunospot) (*16*, *17*). Thus, recently, high-throughput T cell antigen identification assays have been developed to sort immunogenic epitopes from libraries of many thousands of encoded peptides (*18*–*21*). However, these assays are limited by the need to test single TCRs one by one. Eventually, solving the specificities of large numbers of T cells within the immune repertoire, including the set of many thousands of public TCRs (*3*), will require approaches that preserve this highly scaled putative antigen testing, but better scale in TCR numbers.

Here, we sought to identify the target specificities of public TCRs de novo. We developed a workflow, which we refer to as “AIMcap (activation-induced marker–mediated capture of target epitopes)”, in which we first screen a panel of public TCRs for reactivity to hundreds or thousands of peptides in single wells of a 96-well plate; and then, using an activation-induced marker (AIM)–based antigen sorting method, we identify the specific viral peptide(s) targeted within the well.

## RESULTS

### Selection of public TCRs and establishment of a system to screen for viral antigen reactivity

**Fig. 1.**
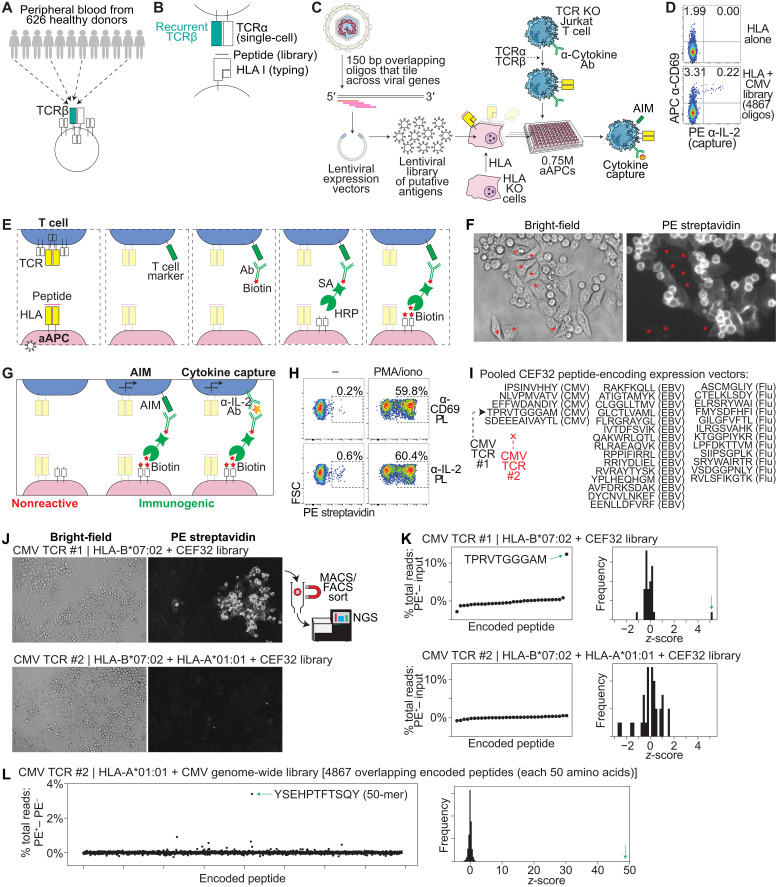
Development of the AIMcap system. (**A**) “Public” TCRβ sequences are found in human samples. (**B**) Schematic of gene sources used to test HLA-peptide-TCR interactions. (**C**) Schematic of viral genome library screening using Jurkat cells expressing an anti-IL-2 capture antibody. Ab, antibody; bp, base pair. (**D**) Flow cytometric analysis (APC anti-CD69 versus PE anti-IL-2) of CMV-TCR #1–expressing Jurkat cells after coculture with HLA-B*07:02^+^ aAPCs ± CMV library. Representative of three independent experiments. (**E**) Schematic of PL assay. (**F**) Bright-field (left) and fluorescence (right) images of aAPC-Jurkat cocultures after anti-CD2–mediated PL. Jurkat cells and interacting aAPCs stained PE^+^; noninteracting aAPCs (asterisks) remained PE^−^. (**G**) Antibodies against AIM markers or captured cytokines can be used to drive PL, enabling activation-dependent staining. (**H**) Flow cytometric analysis [forward scatter (FSC) versus PE-SA] of aAPCs after coculture with IL-2–capture Jurkat cells ± PMA/ionomycin, followed by anti-CD69– or anti-IL-2–mediated PL. Representative of three independent experiments. (**I**) Schematic showing the pooled set of 32 CEF peptides, with CMV-TCR #1 epitope; CMV-TCR #2 does not recognize the library. (**J**) Bright-field (left) and fluorescence (right) images of clonally expanded aAPCs expressing the CEF32 library and respective HLA alleles, cocultured with Jurkat cells expressing CMV-TCR #1 (top) or #2 (bottom). Anti-CD69–mediated PL marked only specific aAPC–Jurkat cells. Labeled aAPCs can be sorted and sequenced. (**K**) Stained cells from (J) were sorted, and epitope-encoding DNA was sequenced. Differences in percent total NGS reads between sorted and unlabeled cells for each encoded peptide (I) are shown (left), with *z*-score distributions (right). Representative of two independent experiments. (**L**) HLA-A*01:01^+^ aAPCs expressing the CMV genome library were cocultured with CMV-TCR #2 Jurkat cells (target: YSEHPTFTSQY), and anti-CD69–mediated PL was performed. Sequence data were graphed similar to (K). Green arrows indicate target peptide.

We evaluated a set of TCRβ sequences that are (i) recurrently found in clonally expanded T cells in the peripheral blood of healthy individuals [in samples collected pre–COVID-19 pandemic; (*1*)], (ii) found in single-cell TCR sequencing datasets along with a paired TCRα gene, and (iii) recurrently found in individuals who share a common human leukocyte antigen (HLA) class I allele [suggesting antigen recognition rather than increased generational likelihood during V(D)J recombination] (Fig. 1, A and B; fig. S1A; and tables S1 to S3) (*3*). A subset of these T cells had evidence of specificity for CMV, EBV, or Flu A, but others have unknown target specificities (table S3) (*3*). Given their recurrent identification in the healthy population, we hypothesized that the unknown TCRs might also recognize common viral antigens. Identifying their specificities could provide insight into infection-induced immune memory in humans. Therefore, we sought to develop a workflow capable of identifying cognate epitopes from thousands of candidates, using overlapping minigenes that tile across viral genes (Fig. 1C), in a rapid and scalable manner.

In the first stage of our workflow, putative antigens are cloned into lentiviral vectors and transduced into patient HLA-compatible “artificial” antigen-presenting cells (aAPCs) (*21*), targeting a multiplicity of infection (MOI) >/= 1 (Fig. 1C). Approximately 7500 aAPCs could be placed into each well of a 96-well plate. We envisioned an initial screen, in which each candidate TCR is cloned into TCR knockout (KO) Jurkat T cells (*21*), and tested for reactivity in these aAPC library–containing wells (Fig. 1C). In this setting, because the target peptide(s) would be expressed by only a small percentage of the aAPCs, the expected result would be the activation of a fraction of the input Jurkat cells (Fig. 1D). We reasoned that this screening stage would allow us to determine whether the TCR is reactive, or not, to a selected antigen library (e.g., to a specific virus) and that we could scale this approach to evaluate a panel of TCRs in a 96-well plate (Fig. 1C).

To test this, we cocultured Jurkat cells expressing CMV-TCR #1 [an HLA-B*07:02–restricted public TCR that is known to target the CMV epitope TPRVTGGGAM; (*1*, *22*)] with aAPCs expressing HLA-B*07:02 alone, or HLA-B*07:02 and a tiled genome-wide CMV library (consisting of 4867 overlapping encoded peptides of 50 amino acids/peptide) (*21*). While staining for surface expression of CD69 (an AIM) has previously been used to detect TCR engagement in Jurkat cells (*6*, *23*, *24*), we observed CD69 expression in the Jurkat cells under the control (HLA only) condition (Fig. 1D and fig. S1B); moreover, for different TCRs, baseline CD69 expression varied for each Jurkat cell we prepared over a >10-fold range (fig. S1C). In the presence of the CMV library, while CD69 expression increased 2.17-fold on average (Fig. 1D and fig. S1B), we sought to improve the specificity of the activation-based readout by additionally measuring interleukin-2 (IL-2) production. Rather than inhibiting IL-2 secretion and then fixing/permeabilizing the cells for intracellular cytokine staining (*25*), we engineered Jurkat cells to express a surface-anchored, high-affinity anti-IL-2 antibody, enabling live Jurkat cells to capture their own secreted IL-2 (Fig. 1C). IL-2 accumulation on the cell surface was then detected using a fluorescently labeled secondary anti-IL-2 antibody. The combination of CD69 (a less specific, but cell-intrinsic activation marker) and captured IL-2 (a more specific, but secreted activation marker) was then quantitated.

In the absence of coculture with the CMV library, we observed negligible CD69^+^IL-2^+^ background staining, while the signal with the 4867 encoded peptide CMV library was readily observable in one well of a 96-well plate (Fig. 1D and fig. S1B; *P* < 0.0005). In addition, we reduced the MOI of the target epitope and observed a quantitative relationship between epitope abundance and Jurkat activation (fig. S1, D to E), with a marked improvement in fold change of the IL-2^+^ signal relative to the CD69^+^ signal alone (fig. S1F). Testing of a nontargeted encoded peptide demonstrated the specificity of the signal (fig. S1G). Notably, a majority (often >85%) of IL-2 capture occurred on CD69^+^ Jurkat cells (Fig. 1D and fig. S1, D and G), suggesting that the activated Jurkat cells capture their own IL-2 preferentially, while only a small fraction of IL-2 may leak to neighboring Jurkat cells. Together, we demonstrate a sensitive and specific dual reporter that could be used to identify TCRs that recognize peptide(s) within highly complex libraries of putative viral antigens.

### Establishing a system for T cell epitope sorting and identification using proximity labeling

In the second stage of our workflow, for each TCR that is reactive in our first stage screen, we sought to pinpoint the specific peptide(s) that is recognized within the library. We reasoned that if we similarly cocultured the TCR-expressing Jurkat cells with the library, but then stained the activated Jurkat cell surface with antibodies linked to horseradish peroxidase (HRP) instead of phycoerythrin (PE) (as in Fig. 1D), we could use HRP’s ability to catalyze proximity labeling (PL) (*26*) to send biotin molecules from the surface of the activated Jurkat cells down to the proximal APCs (Fig. 1E). This would allow us to sort the immunogenic peptides from our input libraries and to pinpoint the peptides by next-generation sequencing (NGS) of the encoded peptides, and would also allow us to use the same endogenous signals of T cell activation, including AIM and cytokine secretion, that we used for screening, to preserve the sensitivity and specificity of these canonical readouts (*17*, *24*, *25*).

As a proof of principle of signal transfer from Jurkat cells to aAPCs using PL, after coculture, we stained Jurkat cells with a biotinylated anti-CD2 antibody [to stain all Jurkat cells, because CD2 is constitutively expressed; (*27*)]. Second, we linked the biotin to a streptavidin-HRP (SA-HRP) fusion protein to coat the Jurkat cell surface with HRP enzyme. Third, we added the substrate biotin-xx-tyramide to enable HRP to covalently biotinylate membrane proteins at the Jurkat cell–aAPC interface (Fig. 1E). We confirmed by imaging that both the Jurkat cells and the interacting aAPCs become biotinylated. aAPCs that were not in contact with Jurkat cells were not visibly biotinylated, consistent with localized signal transfer in a proximity-dependent manner (Fig. 1F).

Next, we sought to determine whether we could turn on PL in a T cell activation–dependent manner. Rather than staining CD2 (which is constitutively expressed on T cells), after coculture in the absence or presence of phorbol 12-myristate 13-acetate (PMA) and ionomycin (which nonspecifically activate T cells), we stained the Jurkat cells with biotinylated antibodies that bind to CD69 or to captured IL-2, which are markedly activation-induced, followed by staining with SA-HRP, biotin-xx-tyramide, and PE-SA. With both anti-CD69 and anti-IL-2 antibodies, we found that aAPCs become labeled with PE-SA preferentially under the PMA/ionomycin condition (59.8 and 60.4% labeling, respectively), with minimal background staining in the absence of PMA/ionomycin (0.2 and 0.6%, respectively) (Fig. 1H). Second, to test for PL in an epitope-specific manner, we cocultured Jurkat cells expressing CMV-TCR #1 or CMV-TCR #2 [an HLA-A*01:01–restricted public TCR that is known to target the CMV epitope YSEHPTFTSQY; (*1*, *21*)] with aAPCs expressing HLA-A*01:01 and HLA-B*07:02; these HLA genes with CMV-TCR #1’s target epitope; or the HLA genes with CMV-TCR #2’s target epitope. We then stained the Jurkat cells with biotinylated anti-CD2, biotinylated anti-CD69, or biotinylated anti-IL-2 antibody and performed our PL method (fig. S1, H to J). Anti-CD2 led to PL in the presence of either the nontarget or target peptide; although with the target peptide, a moderate (1.3- to 2.1-fold) increase in labeling efficiency was seen (fig. S1H), possibly due to increased Jurkat-aAPC contact upon activation (*28*). In contrast, anti-CD69 and anti-IL-2 led to a substantial (28- to 187-fold) difference between the nontargeted and targeted peptides (fig. S1, I and J).

We thus sought to determine whether anti-CD69– and anti-IL-2–mediated PL method could separate Jurkat T cell–activating aAPCs from non-activating aAPCs in a pooled format (i.e., within a dish). As a proof of concept, we transduced a library of 32 CEF (CMV, EBV, and Flu) peptide-encoding oligonucleotides (oligos) (*21*, *29*) into HLA-B*07:02–expressing aAPCs at an MOI of ~1, allowed the aAPCs to expand for several passages, and then cocultured the aAPC library with CMV-TCR #1–expressing Jurkat cells. Following PL using an anti-CD69 antibody, we observed focal areas of staining of aAPCs and Jurkat cells, consistent with recognition of a single epitope within the CEF32 library (Fig. 1, I and J, top). To quantify enrichment of the proximity-labeled aAPCs, we sorted PE^+^ cells from PE^−^ cells using magnetic-activated cell sorting (MACS), extracted genomic DNA from the PE^+^ and PE^−^ cell populations, polymerase chain reaction (PCR)–amplified the peptide-encoding minigenes, and performed NGS. We found that 13.79% of total peptide-encoding reads encoded the CMV-TCR #1 target, TPRVTGGGAM in the PE^+^ (+sort) population (fig. S1K, left). In comparison, TPRVTGGGAM was represented by 1.44% of peptide-encoding reads in the PE^−^ (−sort) population (fig. S1K, left). This difference of 12.35% was the most substantial outlier among the library, with a *z*-score of 5.23 (Fig. 1K, top, and table S5A). Similarly, using anti-IL-2–mediated PL, we observed that the TPRVTGGGAM was the most substantial outlier among the CEF32 library, with a *z*-score of 4.59 (fig. S1L, top, and table S5B). In contrast, using anti-CD2–mediated PL, we did not observe TPRVTGGGAM as an outlier (fig. S1M, top, and table S5C).

The cognate epitope of CMV-TCR #2, in contrast, is absent from the CEF32 library (Fig. 1I). As a negative control, we transduced the CEF32 library into HLA-A*01:01– and HLA-B*07:02–expressing aAPCs, and cocultured them with CMV-TCR #2–expressing Jurkat cells. Following PL using an anti-CD69 antibody, we did not observe the focal areas of staining of aAPCs and Jurkat cells that we observed in the CMV-TCR #1 coculture (Fig. 1J, bottom), but observed only scattered signal consistent with background PL (Fig. 1H, top left). We then sorted PE^+^ from PE^−^ cells using MACS, PCR-amplified the peptide-encoding inserts, and performed NGS. We observed comparable peptide distributions between the PE^+^ and PE^−^ fractions (Fig. 1K, bottom, and table S5D), consistent with the absence of a target peptide. Similarly, anti-IL-2– and anti-CD2–mediated PL resulted in an absence of an enriched peptide (fig. S1, L and M, bottom; and table S5, E and F).

To confirm that we could identify the CMV-TCR #2 target epitope, we transduced our CMV genome-wide library (4867 encoded peptides) into HLA-A*01:01–expressing aAPCs and cocultured the aAPC library with CMV-TCR #2–expressing T cells. After anti-CD69–mediated PL, we found that 3.44% of total peptide-encoding reads contained the CMV-TCR #2 target, a YSEHPTFTSQY-containing 50 amino acid oligomer (50-mer), in the PE^+^ cell population. In comparison, this target was represented by 0.04% of peptide-encoding reads in the PE^−^ population. This difference of 3.40% was the most substantial outlier among the library, with a *z*-score of 48.78 (Fig. 1L and table S5G). Together, these results demonstrated that our assay can identify the cognate T cell–targeted epitope from mixed pools containing tens to thousands of peptide-encoding oligos through PL mediated by either CD69 or surface-bound IL-2.

### Systematic testing of putative antigen libraries for T cell reactivity

To screen for antigen reactivity, we had previously synthesized 4867 peptide-encoding oligos that tile across all annotated genes of CMV (*21*), which has the largest genome among pathogenic human viruses (*30*). Given the high population incidence of EBV infection in the healthy US population (*31*) and the observation that CMV and EBV may represent the most frequent known specificities among public TCRs (*3*), we designed and synthesized 3517 peptide-encoding oligos that tile across all annotated genes from three reference EBV strains. We also synthesized 1266 peptide-encoding oligos that tile across all annotated genes from eight reference Flu A or B viral strains. Each of these oligos encodes a 50–amino acid peptide that overlaps with the adjacent encoded peptide (Fig. 1C). We cloned these oligo libraries into lentiviral expression vectors. We then transduced HLA KO aAPCs (*21*) with our CMV, EBV, or Flu library at an MOI of 5 to 10, along with the respective HLA allele(s) (table S3).

**Fig. 2.**
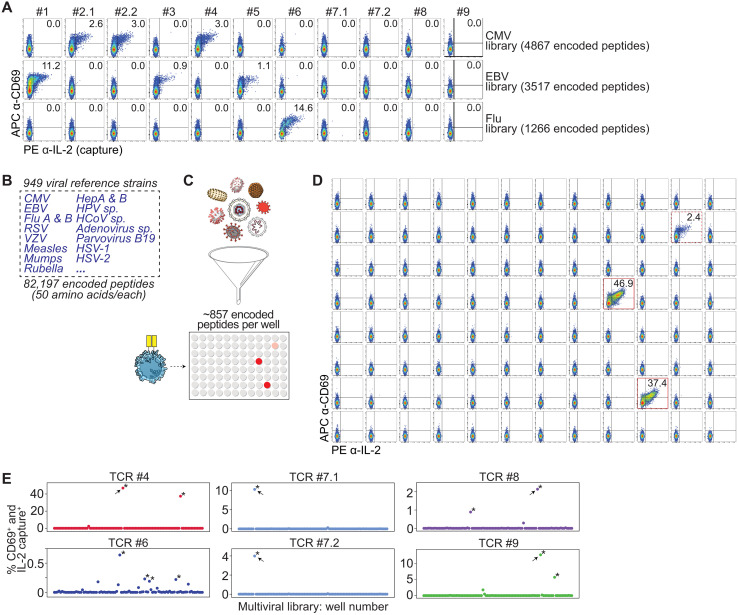
Screening libraries of reference human viruses. (**A**) Flow cytometric analysis of the indicated TCR-expressing Jurkat cells showing APC anti-CD69 versus PE anti-IL-2 (capture) after coculture with aAPCs expressing HLA-A*01:01, HLA-A*02:01, HLA-B*07:02, HLA-B*08:01, and a genome-wide tiled CMV, EBV, or Flu encoded peptide library. (**B**) From 949 viral reference strains, 82,197 overlapping 50–amino acid peptide-encoding oligos were synthesized. (**C**) Schematic showing the 82,197 peptide-encoding genes partitioned into 96 sublibraries of ~857 genes each, which were transduced into aAPCs and cocultured with TCR-transduced Jurkat cells. (**D**) Flow cytometric analysis of TCR #4–expressing Jurkat cells showing APC anti-CD69 versus PE anti-IL-2 (capture) after coculture with aAPCs expressing HLA-A*01:01 and each sublibrary in a 96-well plate. Data are representative of three independent experiments. (**E**) Scatter plot of flow cytometry data plotting CD69^+^IL-2^+^ Jurkat cells in each well after coculture of indicated TCR-expressing Jurkat cells with aAPCs expressing each sublibrary. Arrows point to wells that were used downstream for Jurkat cell PL assays. *z-score > 2. Screens in (A) and (E) were performed once.

As a control, we cloned a public TCR that is known to target the EBV epitope CLGGLLTMV (“TCR #1”) (*32*), and as a discovery set, we cloned 10 additional public TCRα/TCRβ pairs (fig. S1A and table S3) into lentiviral expression vectors; we transduced the lentivirus into our engineered Jurkat cells. We then cocultured each of our TCR-transduced Jurkat cells with the aAPC libraries and measured CD69 surface expression and IL-2 capture by flow cytometry. Given the complexity of these aAPC libraries, we expected that only a subset of the aAPCs express the cognate epitope. Accordingly, when Jurkat cells expressing TCR #1 were cocultured with aAPCs transduced with CMV, EBV, or Flu peptide libraries, 11.2% of the Jurkat cells became dual CD69^+^/IL-2^+^ in response to the EBV library. In contrast, negligible CD69^+^/IL-2^+^ Jurkat cells were observed following coculture with aAPCs presenting the CMV or Flu libraries (Fig. 2A), demonstrating the specificity of the assay.

TCR #2.1 and TCR #2.2 share the same TCRβ sequence but are paired with distinct TCRα chains (table S2). This TCRβ sequence was previously identified through peptide–MHC (pMHC) tetramer sorting using HLA-A*01:01 loaded with the CMV-derived epitope VTEHDTLLY from the UL44 protein (*22*), although the corresponding TCRα chain was not determined in that study. We identified the TCRβ sequence in single-cell sequencing datasets, along with two possible paired TCRα heterodimers (*33*, *34*). Consistent with the tetramer data, we confirmed that both TCRs elicit Jurkat activation in the presence of the CMV library but not the EBV or Flu libraries (Fig. 2A). We then validated that they target the CMV epitope VTEHDTLLY (fig. S2A). Similarly, the TCRβ #3 sequence was previously identified after pMHC tetramer sorting (*35*) but had an unknown TCRα heterodimer. After identification of the paired TCRα gene in a single-cell dataset (table S2), we confirmed that TCR #3 recognizes the EBV epitope RAKFKQLL from the BZLF1 protein (fig. S2B).

For the other TCRs, we could not find tetramer data supporting their target identity (*5*). After Jurkat/aAPC library coculture, we found that one of these public TCRs (TCR #4) was reactive to our CMV library; one public TCR (TCR #5) was reactive to our EBV library; one public TCR (TCR #6) was reactive to our Flu library; and four public TCRs (TCRs #7.1, #7.2, #8, and #9) did not appear to be reactive to our CMV, EBV, or Flu libraries (Fig. 2A). For the CMV-, EBV-, and Flu-library reactive TCRs, we sought to test their reactivity to 32 common CMV, EBV, and Flu encoded peptides [i.e., the CEF32 peptide set; (*21*, *29*)]. We determined that TCR #5 recognizes the EBV epitope RAKFKQLL from the CEF32 peptide set (fig. S2, C and D), but TCRs #4 and #6 did not appear to be reactive to the CEF32 set (fig. S2C).

Together, these results suggested that our workflow could efficiently determine whether a TCR reacts to, or does not react to, a given viral library within a single well of a 96-well plate. Furthermore, we reasoned that if we could test thousands of encoded peptides in each well, we could theoretically screen a single TCR against hundreds of thousands of encoded peptides in a 96-well plate or in a subset of one plate.

### Screening a library of reference human viruses

To identify the target antigens of TCRs #7.1, #7.2, #8, and #9, we expanded our search to additional viral genomes. We thus synthesized 82,197 peptide-encoding oligos that tile across all annotated genes of 949 reference strains of viruses that are known to infect humans (Fig. 2B and table S4) (*36*); each of these oligos encodes a 50–amino acid peptide that overlaps with the adjacent encoded peptide (Fig. 1C).

As peptide library complexity increases, we reasoned that an increasingly smaller number of activated Jurkat cells would be detected. As a test, we cocultured TCR #2.1–expressing Jurkat cells with aAPCs expressing HLA-A*01:01 and (i) the target epitope, (ii) the 4867 encoded peptide CMV library, or (iii) the 82,197 encoded peptide multiviral library. As expected, the CD69^+^/IL-2^+^ signal decreased as library complexity increased (fig. S2E). To improve sensitivity as well as to reduce the complexity of the epitope-containing library, we subdivided the full 82,197-peptide library into 96 smaller sublibraries, each comprising ~857 peptide-encoding oligos. We transduced the sublibraries into HLA-compatible aAPCs in a 96-well plate and then cocultured each Jurkat cell clone with each of the 96 sublibrary wells (Fig. 2C and fig. S2F).

As a test, we used the CMV-reactive TCR #4–expressing Jurkat cell clone and the flu-reactive TCR #6–expressing Jurkat cell clone. For TCR #4, we identified two wells (with *z*-score > 2) that contained activated Jurkat cells (Fig. 2, D and E, and fig. S2C). For TCR #6, we identified four wells (with *z*-score > 2) that contained activated Jurkat cells (Fig. 2E). This was in line with our expectation: Because the peptides are overlapping, the minimal HLA-binding peptide may be found within multiple 50-mer peptides; in addition, similar viral strains may contain the same minimal peptide but with slight differences in other regions of the 50-mer.

Extending this screening approach to our TCRs that were not reactive to CMV, EBV, or Flu (i.e., TCRs #7.1, #7.2, #8, and #9), we found that for each TCR, there was >/= 1 sublibrary that activated the Jurkat cells (Fig. 2E). TCRs #7.1 and #7.2, which share the same TCRβ sequence but are paired with different TCRα heterodimers, gave a similar pattern of subpool reactivity (Fig. 2E and fig. S2G), suggesting that they recognize the same epitope but have allowance for sequence variability of the TCRα heterodimer (*22*). These results suggested that we could narrow antigen specificity within a highly complex multiviral library down to the ~1% that contains the target epitope and that TCRs #7.1, #7.2, #8, and #9 are indeed antiviral TCRs, but target epitopes outside of CMV, EBV, and Flu.

### Determination of the target virus of public TCRs by PL

**Fig. 3.**
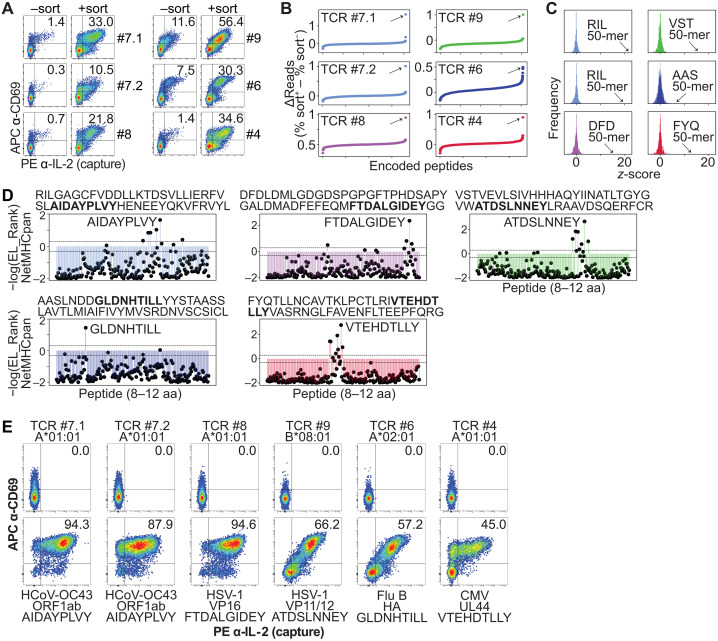
Determination of the target viral epitope using PL. (**A**) Flow cytometric analysis of the indicated TCR-expressing Jurkat cells showing APC anti-CD69 versus PE anti-IL-2 (capture) after coculture with aAPCs that underwent PL and sorting. PL was performed using the respective target viral genome or the multiviral sublibrary, and Jurkat cell clones were cocultured with the unsorted (−sort) or sorted (+sort) aAPC populations, showing antigen enrichment within the sorted population. Experiment was performed once. (**B**) Difference between sorted and unsorted cells in percent total reads for each encoded peptide sequence after PL. Arrows point to the top candidate. Screens were performed once. (**C**) Frequency of *z*-score measurements from screening data in (B). Arrows point to the top candidate 50-mer encoded peptide. (**D**) %EL_Rank scores (−log) from NetMHCpan-4.1 for candidate 50-mer encoded peptide in (C). Thresholds for strong binders (SB) and weak binders (WB) are marked. aa, amino acids. (**E**) Flow cytometric analysis of indicated TCR-expressing Jurkat cells showing APC anti-CD69 versus PE anti-IL-2 (capture) after coculture with aAPCs expressing the indicated restricting HLA allele without or with the candidate minimal epitope in (D). Data in (E) are representative of three independent experiments. HCoV, human coronaviruses.

Using PL, we sorted the immunogenic encoded peptides starting with either the single virus library (Fig. 2A) or the most reactive sublibrary in the multiviral screen (Fig. 2E). After coculture, staining, and aAPC sorting, we aliquoted a fraction of the +sorted and −sorted aAPCs back into cell culture. After multiple passages, we added fresh TCR-transduced Jurkat cells to these aAPC aliquots and found that the +sorted aAPCs caused increased Jurkat cell activation compared to the −sorted aAPCs (Fig. 3A), consistent with enrichment of the target epitope within the +sorted aAPCs. We PCR-amplified the encoded peptides within the +sorted and −sorted aAPCs, performed NGS, and determined the encoded peptide sequence(s) enriched in the +sorted aAPCs.

For both TCRs #7.1 and #7.2, using the most reactive sublibrary (Fig. 2E), we identified a 50-mer (referred to here as the “RIL 50-mer” sequence) from seasonal coronavirus HCoV-OC43 as the top hit (Fig. 3B; fig. S3A; and table S5, H and I), with *z*-scores of 21.97 and 19.79, respectively (Fig. 3C). The TCRβ #7.1 sequence is found in the peripheral blood of 14.4% (90/626) of individuals within the cohort [drawn from healthy donors in the US; (*1*)], and 46.2% of the HLA-A*01:01^+^ individuals (fig. S1A and table S1), consistent with the high (>90%) exposure rate of healthy individuals to seasonal coronavirus HCoV-OC43, which causes the common cold (*7*, *37*).

For TCR #8, using the most reactive sublibrary (Fig. 2E), we identified a 50-mer (referred to here as the “DFD 50-mer” sequence) from HSV-1 as the top hit (Fig. 3B and table S5J), with a *z*-score of 15.65 (Fig. 3C). The TCRβ #8 sequence is found within 5.0% (31/626) of the cohort, and 14.5% of the HLA-A*01:01^+^ individuals (fig. S1A and table S1) (*1*), consistent with the ~48% exposure rate of healthy individuals to HSV-1 (*8*). For TCR #9, using the most reactive sublibrary (Fig. 2E), we identified a 50-mer (referred to here as the “VST 50-mer” sequence) from HSV-1 as the top hit (Fig. 3B and table S5K), with a *z*-score of 16.08 (Fig. 3C). The TCRβ #9 sequence is found within 5.0% (31/626) of the cohort, and 15.6% of the HLA-A*01:01^+^ individuals (fig. S1A and table S1) (*1*).

For TCR #6, using the Flu library (Fig. 2A), we identified a 50-mer (referred to here as the “AAS 50-mer” sequence) within Flu B as the top hit (Fig. 3B and table S5L), with a *z*-score of 6.48 (Fig. 3C). The TCRβ #6 sequence is found within 5.1% (32/626) of the cohort, and 10.9% of the HLA-A*02:01^+^ individuals (fig. S1A and table S1) (*1*). For TCR #4, using the most reactive sublibrary (Fig. 2E), we identified a 50-mer (referred to here as the “FYQ 50-mer” sequence) from CMV as the top hit (Fig. 3B and table S5M), with a *z*-score of 13.47 (Fig. 3C). The TCRβ #4 sequence is found within 5.3% (33/626) of the cohort, and 10.2% of the HLA-A*01:01^+^ individuals (fig. S1A and table S1) (*1*).

Together, we identified viral targets for each of the public TCRs, including those with previously unknown target identity despite their common frequencies within healthy individuals. Our results revealed common viral targets apart from CMV, EBV, and Flu A, thus providing a workflow to broaden existing knowledge of public TCR target specificities.

### Determination of complete TCR-peptide-HLA complexes

We then sought to molecularly define the complete TCR-peptide-HLA complexes containing each of the public TCRs. Because the libraries were screened using encoded peptides of 50 amino acids each, we predicted the minimal HLA-binding peptides using NetMHCpan-4.1 (*38*) and then functionally validated these peptide sequences.

For the HCoV-OC43-reactive TCRs #7.1 and #7.2, we functionally validated the RNA-dependent RNA polymerase [non-structural protein 12 (NSP12)]–derived peptide AIDAYPLVY, which was the top ranked peptide within the RIL 50-mer predicted to bind to HLA-A*01:01 (Fig. 3, D and E, and fig. S3, A and B). For the HSV-1–reactive TCR #8, we validated the alpha integrating protein [virion protein 16 (VP16)]–derived peptide FTDALGIDEY, which was the top ranked peptide within the DFD 50-mer predicted to bind to HLA-A*01:01 (Fig. 3, D and E). For the HSV-1–reactive TCR #9, we validated the VP11/12-derived peptide ATDSLNNEY, which was the top ranked peptide within the VST 50-mer predicted to bind to HLA-A*02:01 (Fig. 3, D and E). For the Flu-reactive TCR #6, we validated the Flu B hemagglutinin (HA)–derived peptide GLDNHTILL, which was the top ranked peptide within the AAS 50-mer predicted to bind to HLA-A*02:01 (Fig. 3, D and E). For the CMV-reactive TCR #4, we validated the UL44-derived peptide VTEHDTLLY, which was the top ranked peptide within the FYQ 50-mer predicted to bind to HLA-A*01:01 (Fig. 3, D and E).

After demonstrating that the public TCRs identified in this study are reactive to viral epitopes, we next asked whether CD8^+^ T cell responses to these epitopes can be detected in the blood of healthy individuals. To test this, we stimulated CD8^+^ T cells isolated from the peripheral blood of six HLA-A*01:01^+^ or HLA-A*02:01^+^ healthy donors with our identified epitopes, and measured aAPC interferon-γ (IFN-γ) capture (*21*). Despite this small sample size, we observed CD8^+^ T cell responses against AIDAYPLVY, FTDALGIDEY, GLDNHTILL, and VTEHDTLLY (fig. S3C). These responses are consistent with these epitopes being commonly targeted by CD8^+^ T cells and further suggest that identifying the antigen specificities of public TCRs can help decipher an individual’s infection history. Together, our workflow can identify complete TCR-peptide-HLA complexes starting from TCRs of unknown target identity within the healthy T cell repertoire.

### Determination of cross-reactivity between viruses and species

**Fig. 4.**
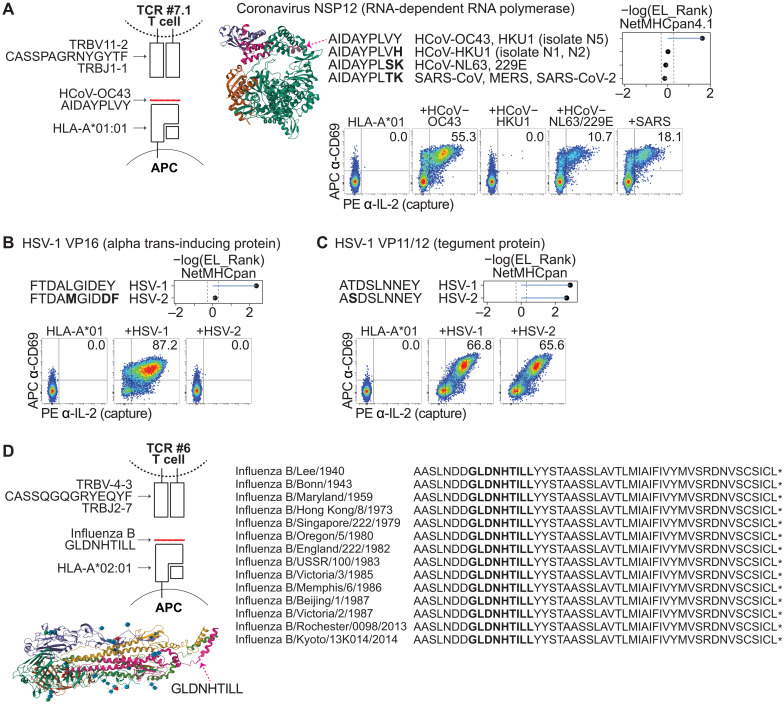
Determination of viral cross-reactivity. (**A**) Schematic representation of the complete TCR-peptide-HLA complex for TCR #7.1. Crystal structure of coronavirus NSP12 taken from Protein Data Bank (PDB) 6NUR; arrow points to the minimal epitope region. The coronavirus HCoV-OC43 epitope as well as the corresponding peptides within HCoV-HKU1, HCoV-NL63/HCoV-229E, and pandemic coronaviruses are shown, along with their %EL_Rank scores (−log) from NetMHCpan-4.1. Flow cytometric analysis of TCR #7.1–expressing Jurkat cells showing APC anti-CD69 versus PE anti-IL-2 (capture) after coculture with aAPCs expressing HLA-A*01:01 without or with the panel of coronavirus peptides. (**B**) The TCR #8 HSV-1 epitope as well as the corresponding peptide within HSV-2 are shown, along with their %EL_Rank scores (−log) from NetMHCpan-4.1. Flow cytometric analysis of TCR #8–expressing Jurkat cells showing APC anti-CD69 versus PE anti-IL-2 (capture) after coculture with aAPCs expressing HLA-A*01:01 without or with the panel of HSV peptides. (**C**) The TCR #9 HSV-1 epitope as well as the corresponding peptide within HSV-2 are shown, along with their %EL_Rank scores (−log) from NetMHCpan-4.1. Flow cytometric analysis of TCR #9–expressing Jurkat cells showing APC anti-CD69 versus PE anti-IL-2 (capture) after coculture with aAPCs expressing HLA-A*01:01 without or with the panel of HSV peptides. Data in (A) to (C) are representative of three independent experiments. (**D**) Schematic representation of the complete TCR-peptide-HLA complex of TCR #6. Crystal structure of Flu HA taken from PDB 6HJR; arrow points to the minimal epitope region. The minimal epitope sequences within the HA C terminus from different Flu B strains are shown.

We then sought to determine the extent of cross-reactivity of our TCRs against closely related viruses and strains. First, we aligned the HCoV-OC43 epitope, AIDAYPLVY, with the corresponding peptides within other seasonal coronaviruses (HCoV-HKU1, HCoV-NL63, and HCoV-229E) as well as within pandemic coronaviruses [SARS-CoV, Middle East respiratory syndrome (MERS), and SARS-CoV-2]. These peptides—AIDAYPLDH, AIDAYPLSK, and AIDAYPLTK—were all predicted by NetMHCpan-4.1 to be weak binders to HLA-A*01:01, in contrast to the HCoV-OC43 peptide, which was predicted to be a strong binder (Fig. 4A). Testing of these peptides showed that the HCoV-NL63/HCoV-229E (AIDAYPLSK) and SARS-CoV-2 (AIDAYPLTK) peptides were able to activate TCR #7.1, although the activation was reduced compared to the HCoV-OC43 epitope (AIDAYPLVY), whereas the HCoV-HKU1 (isolate N1, N2) (AIDAYPLVH) peptide did not activate or very weakly activated this TCR (Fig. 4A and fig. S4A). To confirm that these epitopes could be processed from the open reading frame (ORF), we transduced the HCoV-OC43 NSP12 ORF or the SARS-CoV-2 NSP12 ORF into aAPCs, treated the cells with IFN-γ, and cocultured them with TCR #7.1–expressing Jurkat cells. We found that TCR #7.1 was activated by both the HCoV-OC43 and SARS-CoV-2 NSP12 ORFs (fig. S4B).

Similarly, we aligned the HSV-1 epitopes targeted by TCR #8 and TCR #9 with the corresponding peptides within HSV-2. For TCR #8, the HSV-1 peptide, FTDALGIDEY, was predicted by NetMHCpan-4.1 to be a strong binder to HLA-A*01:01, whereas the corresponding HSV-2 peptide, FTDAMGIDDF, was predicted to be a weak binder (Fig. 4B). For TCR #9, both the HSV-1 and HSV-2 peptides, ATDSLNNEY and ASDSLNNEY, were predicted to be strong binders to HLA-A*01:01 (Fig. 4C). Testing of FTDAMGIDDF suggested that TCR #8 (fig. S4C) did not cross-react with its counterpart in HSV-2 (Fig. 4B). In contrast, testing of ASDSLNNEY suggested that TCR #9 (fig. S4C) is cross-reactive with its counterpart in HSV-2 (Fig. 4C).

Last, we aligned the Flu B epitope, GLDNHTILL, with the corresponding peptide within the strains of Flu B represented in our library. Notably, we did not identify any sequence variability between the strains (Fig. 4D). While the head and stalk regions of Flu HA are known to be particularly variable between strains as they are common targets of neutralizing antibodies (*39*), the GLDNHTILL epitope is in the junction of the flexible linker region and the transmembrane domain (Fig. 4D), which may be less commonly subjected to antibody-mediated evolution (*40*), and may represent a common Flu B epitope in the population (fig. S3C) (*40*, *41*).

Together, these results suggested that TCR #7.1 may be cross-reactive to other coronaviruses, including pandemic coronaviruses that emerged prospectively; TCR #8 appears specific to HSV-1, while TCR #9 appears to recognize both HSV-1 and HSV-2; and TCR #6 appears to target all, or nearly all, Flu B strains.

## DISCUSSION

We describe a complete workflow to decipher public TCR reactivities de novo: (i) Using our engineered dual reporter Jurkat cells, we demonstrate that TCRs can be rapidly tested against complete viral genomes in a single well of a 96-well plate, or against ~1000 viral genomes (using 82,197 overlapping 50–amino acid encoded peptides) within one plate; and (ii) using PL, we demonstrate that we can pinpoint each TCRs’ target epitope specificities. Using our AIMcap workflow, we identified specificities of previously “orphan” TCRs that are commonly circulating in healthy individuals. These TCRs are found in 3.5 to 14.4% of a US cohort (table S1), suggesting that >10 million individuals in the US have each of these TCRs in circulation. As hypothesized, we identified TCR specificities beyond common epitopes within CMV, EBV, and Flu A (fig. S4D). For example, despite its high frequency as a T cell target, the seasonal (“common cold”) coronavirus epitope AIDAYPLVY was not previously found in existing epitope databases (*5*, *42*), nor were there databased examples of many of our TCR-peptide-HLA complexes including our HCoV-OC43, HSV-1, and Flu B–reactive TCRs (*5*). Thus, we demonstrated that our strategy could successfully uncover public TCR specificities without starting with an a priori epitope panel.

Our AIMcap workflow enables efficient prescreening of a panel of T cells for viral genome reactivity, prior to epitope identification. In combination with recently developed high-throughput TCR cloning approaches (*43*), potentially thousands of TCRs could be tested in our workflow against the genomes of CMV and EBV, which may be the most common public TCR specificities (*3*). Furthermore, single viral genome libraries of other common public TCR specificities (e.g., other Herpesviridae or seasonal coronaviruses) could be synthesized and tested. To identify the underlying immunogenic aAPCs, we developed a T cell antigen identification technique based on PL using HRP (*26*) to label the aAPCs that present immunogenic peptide(s) to the TCRs. While other high-throughput antigen identification technologies have been described (*18*–*21*, *44*), T cell PL is the first assay that initiates the signal from T cell cytokine capture or from an AIM signal, which are well-established endogenous signals of functional activation (*17*, *24*, *25*, *45*). Because cytokine secretion and AIM induction are not unique to CD8^+^ T cells, T cell PL should be readily modifiable to study CD4^+^ TCR specificities. Furthermore, the signal in T cell PL is generated using exogenous staining reagents that could be used without aAPC or T cell genetic engineering, and the HRP-linked antibody could readily be swapped out to bind to different targets (Fig. 1G).

Alternative methods to determine TCR target specificity include pMHC multimers (*16*, *41*) and TCR clustering algorithms (*22*, *46*, *47*). Although these are powerful tools, they require a priori knowledge of pMHCs in their routine use. A combined approach that first uses our high-throughput workflow to identify immunodominant peptides to broaden epitope databases beyond CMV, EBV, and Flu pMHCs (fig. S4D), followed by the development of pMHC multimers, their deployment to sort and sequence additional circulating TCRs, and the analysis of the sequencing results using TCR clustering may be the most efficient strategy to solve large numbers of public TCR specificities, as well as to further validate specificities identified by AIMcap. Around 16,951 public HLA-associated TCRs have been enumerated, of which only a small fraction have known target identities (*3*). Gaining a more complete understanding of public TCR specificities has previously enabled the detection of past exposure to certain viruses with high sensitivity and specificity through deep TCR sequencing of peripheral blood samples (*1*, *2*, *13*). A more complete database of public TCR specificities may thus allow diagnosis of past exposure to a large number of antigens concurrently (*2*). Moreover, because certain viral exposures are linked to other acute/chronic diseases, including the association of Flu with Guillain-Barré syndrome (*48*) and the association of EBV with multiple sclerosis (*9*, *10*), identification of antigen-specific public TCRs may help shed light on these associations using patient cohort studies.

We identified a public seasonal coronavirus-reactive TCR that appeared to be cross-reactive to pandemic coronaviruses that emerged prospectively after sample collection, suggesting that this TCR could have contributed to preexisting immunity to pandemic coronaviruses (*37*, *49*, *50*). However, while the HCoV-OC43 peptide has strong NetMHC-predicted binding affinity, the corresponding SARS-CoV-2 peptide has weak predicted binding affinity to HLA-A*01:01 (Fig. 4A), suggesting that the physiological dose of the viral peptide within cells may influence its rate of recognition by T cells.

Along this same vein, despite the large potential space of HLA-binding peptides from each of these viruses (fig. S3B), several of the peptides that we identified through viral genome-wide screening have been repeatedly reported in databases (*5*, *29*, *42*). Our data are consistent with the hypothesis that many of these often-studied epitopes are not just overrepresented in databases (*5*) due to ascertainment bias, but are at the top of viral immunodominance hierarchies (*51*, *52*). Immunodominance is believed to be associated with strong HLA binding affinity, although a more complete model incorporates additional factors that make an epitope more “presented” (i.e., more likely to be seen by and activate a TCR in an individual) than other peptides (e.g., the kinetics of viral protein expression, viral latency states, and viral load) (*51*, *52*). In line with this model, we found that several of the epitopes that we identified, including the coronavirus and HSV-1 epitopes, are not just NetMHC-predicted strong HLA binders, but are among the highest ranked predicted strong binders within their respective virus (fig. S3B). By extension, due to immunodominance, the discovery of public viral epitopes within other human viruses may saturate without the need to screen all public HLA-associated TCRs, i.e., the universe of highly public viral epitopes is likely a finite, discoverable set. Furthermore, the public nature of our Flu B–reactive TCR (TCR #6) may relate to the conservation of its target epitope between strains, i.e., there may be a higher event rate of exposure to conserved epitopes. By extension, other conserved Flu epitopes may be identifiable through public TCR target identification, which may aid in vaccine design (*40*).

Together, we demonstrate that T cell reactivity screening followed by T cell PL is an efficient way to reveal T cell memories de novo. Our overall workflow is agnostic to whether the TCR is public or private; is likely modifiable (*21*) to study CD4^+^ T cell specificities; and may not be limited to studying viral specificities, i.e., an analogous system (*21*) could be envisioned using bacterial peptides (*53*), autoimmune/alloimmune peptides (*10*), tumor peptides (*12*), and/or food/allergen peptides (*54*). Knowledge of TCR-peptide-HLA complexes is increasingly being used to develop diagnostics (*1*, *2*, *13*) and therapeutics (*55*, *56*) such as vaccines and TCR-based cellular therapies (*14*, *15*), underscoring the growing need for efficient strategies to solve HLA-peptide-TCR complexes de novo, as described here.

## MATERIALS AND METHODS

### Experimental design

The goal of this study was to test public TCRs for reactivity to viral genomes to identify their specificities without an a priori epitope panel. To do this, we developed a functional screening workflow in which we synthesize libraries of thousands or tens of thousands of putative antigens, screen panels of TCRs for reactivity to these libraries using an engineered dual reporter Jurkat T cell, and then sort out the immunogenic peptides from each reactive library by labeling APCs that are in proximity to our activated Jurkat T cells. We screened TCRs with known specificity as proof of principle and then screened public TCRs of unknown target specificity.

### Cells, viruses, and reagents

Jurkat cells were obtained from American Type Culture Collection and maintained in RPMI (Gibco) supplemented with 10% fetal bovine serum (FBS; Gemini Bio-Products) and penicillin-streptomycin (Gibco). HeLa and 293T cells were maintained in Dulbecco’s modified Eagle’s medium (DMEM; Gibco) supplemented with 10% FBS. Self-inactivating minimal HIV-1 virus was produced in 293T cells using the vectors pLX301 or pLX303 (Broad Institute, Addgene plasmids #25895 and #25897), the packaging construct psPAX2, and the envelope plasmid pCMV-VSVG. Recombinant IFN-γ was obtained from R&D and added to aAPCs at 12.5 ng/ml. Staining reagents were obtained from the following sources: PE-conjugated anti-IL-2 antibody (N7.48 A, Miltenyi), APC-conjugated and biotin-conjugated anti-CD69 antibody (FN50, BioLegend), PE-conjugated anti-IFN-γ antibody (B27, BioLegend), biotin-conjugated anti-IL-2 antibody (B33-2, BD Biosciences), biotin-conjugated anti-CD2 antibody (RPA-2.10, BioLegend), SA-HRP (BioLegend), biotin-xx-tyramide (SML3484, Sigma-Aldrich; or B40951, Thermo Fisher Scientific), PE-SA (Thermo Fisher Scientific), and Zombie NIR viability dye (BioLegend).

### Public TCR selection, cloning, and expression

TCRβ sequences that were clonally expanded (defined as a frequency > 0.001) in peripheral blood of >1 healthy donor were identified in public TCRβ sequencing data (*n* = 626 donors with HLA class I typing) (*1*). TCRβ sequences that were recurrently found in donors typing for the same HLA allele were selected. A subset of these TCRβ sequences were re-identified in single-cell TCR sequencing datasets from solid tumors (*33*, *34*, *40*, *57*–*59*), which allowed putative identification of the paired TCRα subunit. Paired TCRα and TCRβ sequences were cloned into pLX301 or pLX303. The TCR constant regions (*TRAC* and *TRBC1*) were modified with TRAC p.T48C and TRBC p.S57C mutations to facilitate TCR pairing (*21*, *60*). TCR KO Jurkat T cells (*21*) were transduced with *CD8A* and *CD8B* cDNAs; *LTBR* and *CARD11 p.D357N* cDNAs (*61*, *62*); and an anti-IL-2 antibody (clone NARA1) (*63*) fused to a transmembrane domain as previously described (*21*). These engineered Jurkat cells were then cotransduced with lentivirus that contained the TCRα and TCRβ genes. Cloned TCR sequences are listed in table S3.

### Viral library construction

The CMV library was previously described (*21*). To construct the EBV library, we downloaded sequence data for all ORFs that were annotated in the complete genome sequence of EBV strains AG876 and YCCEL1 in NCBI (accessions NC_009334 and AP015016). We added sequence data for all 92 ORFs that were annotated in the reference proteome of EBV strain B95-8 in UniProt (proteome ID UP000153037). To construct the Flu A/B library, we added sequence data for all ORFs that were annotated in the reference proteome of six Flu A strains (two of H1N1, three of H3N2, and one of H7N7) and two Flu B strains in UniProt (proteome ID UP000009255, UP000171580, UP000096247, UP000166681, UP000115734, UP000123725, UP000099553, and UP000099653). To construct the multiviral library, we downloaded all ORFs that were annotated in the UniProt database (*36*) with “Virus host” = “*Homo sapiens* (Human/Man) [9606]” and “Reviewed” = “Yes.” To construct the EBV and Flu tiled libraries, we tiled all encoded proteins with 50–amino acid peptides, each with a 32–amino acid overlap from the adjacent peptide, and added the C-terminal 50-mer. In total, this resulted in 3517 and 1266 unique peptides, respectively, after filtering out duplicates. To construct the multiviral library, we tiled all encoded proteins with 50–amino acid peptides, each with a 25–amino acid overlap from the adjacent peptide, and added the C-terminal peptide. In total, this resulted in 82,197 unique peptides, after filtering out duplicates, from 949 viral strains. The peptide sequences were converted into DNA sequences, and PCR primer sequences were added to the 5′ and 3′ ends. The DNA libraries were synthesized as oligo pools (Twist Bioscience). Encoded peptides were cloned 3′ of a methionine and a spacer sequence (encoding HTVGLYM between the methionine and the encoded peptide to facilitate our cloning approach) into pLX301 using Gibson Assembly (New England BioLabs). For the multiviral library, sublibraries of ~857 DNA vectors/pool were propagated.

To express minimal peptide epitopes, peptide-encoding oligos (Yale Keck Oligo Synthesis Resource) were cloned in-frame 3′ of the human IL-2 signal sequence (*21*) and assembled into the lentiviral vector pLX301 using Gibson Assembly. To express coronavirus ORFs, the *NSP12* sequences from the encoded ORF1ab polyprotein of coronavirus HCoV-OC43 [UniProt A0A0G2RC73 (amino acids 10 to 928)] or SARS-CoV-2 [UniProt P0DTD1 (amino acids 4407 to 5324)] were synthesized (Twist Bioscience) and cloned 3′ of a methionine into the lentiviral vector pLX301 using Gibson Assembly.

### Coculture and flow cytometry

HLA class I KO (HLA KO) HeLa cells (*21*) were used as aAPCs. aAPCs were cotransduced with HLA cDNAs (HLA-A*01:01, HLA-A*02:01, HLA-B*07:02, and/or HLA-B*08:01), along with peptide-encoding minigenes. For peptide-encoding gene libraries, an MOI of 5 to 10 was targeted [to estimate MOI, we produced lentivirus encoding enhanced green fluorescent protein (eGFP) in parallel with the libraries and used the eGFP infection rate as a reference], with the exception of the CEF32 library in which an MOI of 1 was targeted. Transduced aAPCs were cocultured with TCR-transduced Jurkat cells at an effector:target ratio of ~10:1 (i.e., ~75,000 Jurkat cells to 7500 aAPCs per well) in 96-well plates for 24 hours at 37°C.

For cocultures of aAPCs with primary CD8^+^ T cells, aAPCs were first modified to stably express an IFN-γ capture antibody (clone NI-0501) (*64*) fused to a transmembrane domain (*21*), and then were cotransduced with an HLA cDNA with or without a minimal encoded peptide. CD8^+^ T cells were isolated (Dynabeads Untouched Human CD8 T Cells Kit; Thermo Fisher Scientific) from peripheral blood mononuclear cells that were HLA class I typed (STEMCELL). The CD8^+^ T cells were cocultured with transduced aAPCs at an effector:target ratio of ~10:1 for 24 hours at 37°C. For flow cytometry, after coculture, cells were incubated with indicated antibodies for 30 min at room temperature, washed three times with phosphate-buffered saline (Gibco), and analyzed by flow cytometry (BD CytoFLEX).

### T cell PL assay and immunofluorescence

For library screens, >1000× representation of each encoded peptide within the aAPCs was targeted. After aAPC/Jurkat coculture (as described above), cells were stained with biotin-conjugated anti-CD69, anti-IL-2, or anti-CD2 antibodies for 30 min at 21°C. Jurkat cells were observed to be naturally adherent to the aAPCs, although minimizing cell-cell disruption required avoidance of rigorous pipetting, i.e., solutions were slowly pipetted down the well walls, and after complete removal of the media and a first wash with DMEM, a thin layer of liquid was left on the cells during subsequent (three additional) wash steps. After antibody removal, cells were incubated with SA-HRP (1:100) for 30 min to link the biotinylated antibodies with SA-HRP and then carefully washed to remove excess enzyme. Cells were then incubated with 1 μM biotin-xx-tyramide and 1 mM hydrogen peroxide (Sigma-Aldrich) for 10 to 60 min to enable proximity ligation. Ten-minute incubation resulted in aAPC biotinylation and preserved the ability to further culture the aAPCs, while 60-min incubation resulted in increased aAPC biotinylation but resulted in an inability for the aAPCs to subsequently divide. Cells were then washed, dissociated with 0.25% trypsin-EDTA (Thermo Fisher Scientific), pooled, and labeled with PE-SA, APC anti-CD69 antibody, and Zombie NIR viability dye. Alternatively, the cells were stained with PE-SA before trypsinization and imaged (Bio-Rad ZOE) to visualize fluorescence. PE^+^ aAPCs were sorted from unlabeled cells using a MACS Separator (Miltenyi) with anti-PE MicroBeads (Miltenyi), or using a fluorescence-activated cell sorter (Sony MA900) [an example of the PE^+^ gate (+sort) is shown in fig. S1I]. Aliquots of sorted aAPCs were optionally placed back into a cell culture plate for expansion and repeat of coculture to functionally assess for enrichment of the target antigen.

### Amplification of encoded peptides and sequencing

Genomic DNA was extracted (DNeasy Blood and Tissue Kit, QIAGEN) from PE-labeled cells (+sort) and from unlabeled cells (−sort). The integrated peptide-encoding DNA was amplified by PCR (Kapa HotStart ReadyMix, Roche) using barcoded primers complementary to sequences flanking the peptide-encoding sequences. NGS libraries were prepared from the PCR products and sequenced (Azenta Life Sciences).

### Data analysis

For each NGS read, the barcodes were identified to demultiplex the reads into associated samples, e.g., +sorted cells and −sorted cells. For each read, common sequences flanking the peptide-encoding sequence were identified, and the intervening peptide-encoding sequences were enumerated for each sample. For each sample, the fractional abundance of each encoded peptide was calculated. The difference in fractional abundance of each encoded peptide between pulldown and flow-through samples (annotated as % enrichment) was calculated and graphed. Peptide-HLA binding predictions were performed using NetMHCpan-4.1 (*38*) using default parameters, including EL_Rank thresholds for strong binding (SB) peptides (0.5%) and weak binding (WB) peptides (2%).

### Statistical analysis

Data are reported as mean ± standard deviation. *P* values were calculated using Student’s *t* test (*P < 0.05, **P < 0.005, and ***P < 0.0005). *z*-scores were calculated for encoded peptides identified in the +sort or −sort samples as the difference of each % enrichment value from the mean, divided by the standard deviation.
