# Supplementary material for: De novo identification of the specificities of recurrently identified human T cell receptors
Source: Sci Adv. 2026 Feb 13;12(7):eaeb1732. doi: 10.1126/sciadv.aeb1732 (PMC12904180; doi:10.1126/sciadv.aeb1732)
Supplement: Supplementary file 1 — Figs. S1 to S4 Legends for tables S1 to S5 [file sciadv.aeb1732_sm.pdf]

Science Advances
AAAS

Supplementary Materials for

De novo identification of the specificities of recurrently identified human T cell receptors

Mithila Kasbe *et al.*

Corresponding author: Mark N. Lee, mark.n.lee@yale.edu

Sci. Adv. **12**, eaeb1732 (2026)
DOI: 10.1126/sciadv.aeb1732

The PDF file includes:

Figs. S1 to S4
Legends for tables S1 to S5

Other Supplementary Material for this manuscript includes the following:

Tables S1 to S5

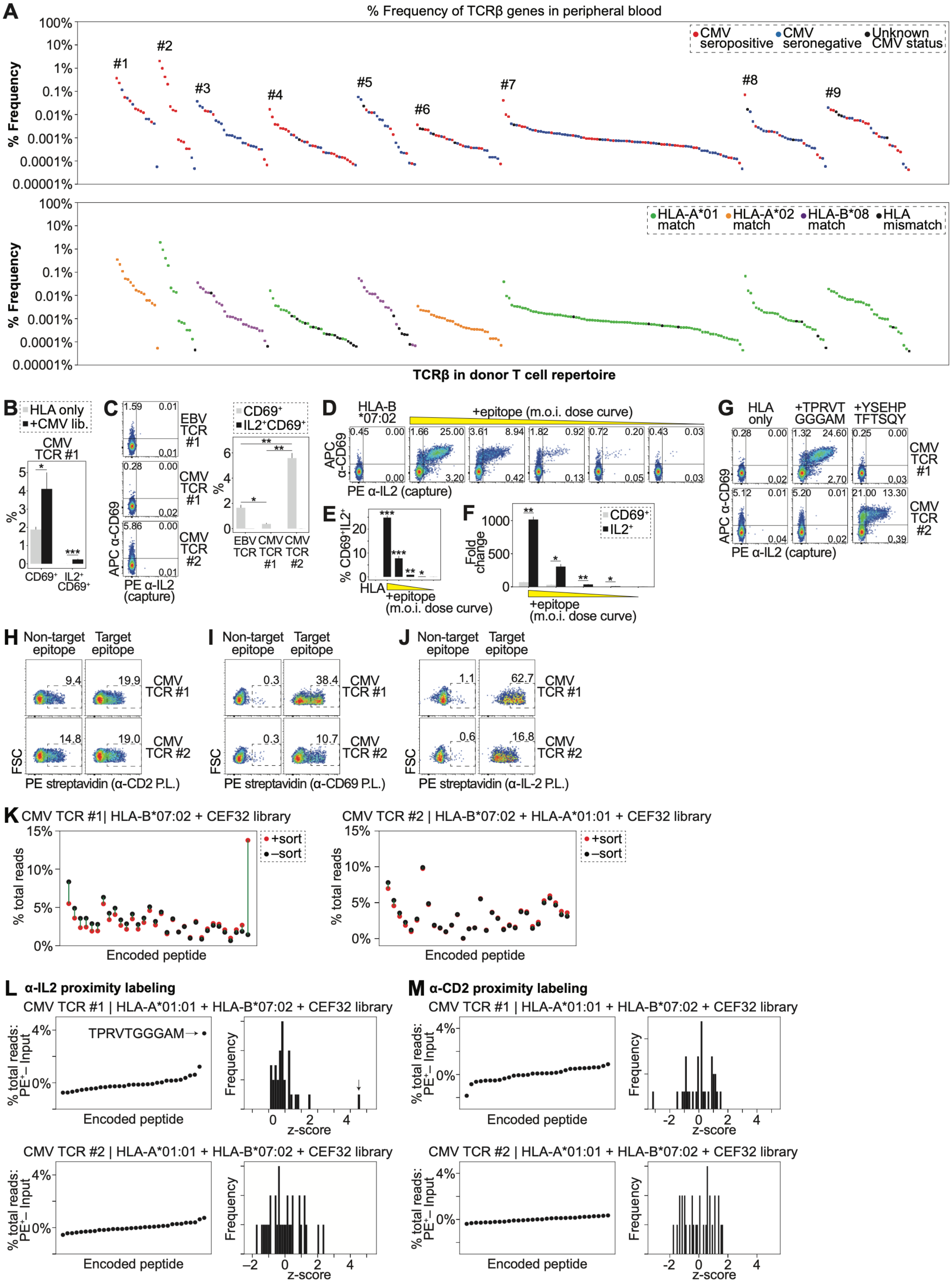
A
% Frequency of TCRβ genes in peripheral blood
CMV seropositive
CMV seronegative
Unknown CMV status
HLA-A*01 match
HLA-A*02 match
HLA-B*08 match
HLA mismatch
% Frequency
100%
10%
1%
0.1%
0.01%
0.001%
0.0001%
0.00001%
#1
#2
#3
#4
#5
#6
#7
#8
#9
TCRβ in donor T cell repertoire
B
HLA only
+CMV lib.
CMV TCR #1
CD69+
IL2+ CD69+
C
EBV TCR #1
CMV TCR #1
CMV TCR #2
APC α-CD69
PE α-IL2 (capture)
CD69+
IL2+CD69+
EBV TCR
CMV TCR #1
CMV TCR #2
D
HLA-B *07:02
+epitope (m.o.i. dose curve)
APC α-CD69
PE α-IL2 (capture)
E
% CD69+IL2+
HLA
+epitope (m.o.i. dose curve)
F
Fold change
CD69+
IL2+
+epitope (m.o.i. dose curve)
G
HLA only
+TPRVT GGGAM
+YSEHP TFTSQY
CMV TCR #1
CMV TCR #2
APC α-CD69
PE α-IL2 (capture)
H
Non-target epitope
Target epitope
CMV TCR #1
CMV TCR #2
FSC
PE streptavidin (α-CD2 P.L.)
I
Non-target epitope
Target epitope
CMV TCR #1
CMV TCR #2
FSC
PE streptavidin (α-CD69 P.L.)
J
Non-target epitope
Target epitope
CMV TCR #1
CMV TCR #2
FSC
PE streptavidin (α-IL-2 P.L.)
K
CMV TCR #1| HLA-B*07:02 + CEF32 library
CMV TCR #2 | HLA-B*07:02 + HLA-A*01:01 + CEF32 library
+sort
–sort
% total reads
Encoded peptide
L
α-IL2 proximity labeling
CMV TCR #1 | HLA-A*01:01 + HLA-B*07:02 + CEF32 library
TPRVTGGGAM→
CMV TCR #2 | HLA-A*01:01 + HLA-B*07:02 + CEF32 library
% total reads: PE+ – Input
Encoded peptide
Frequency
z-score
M
α-CD2 proximity labeling
CMV TCR #1 | HLA-A*01:01 + HLA-B*07:02 + CEF32 library
CMV TCR #2 | HLA-A*01:01 + HLA-B*07:02 + CEF32 library
% total reads: PE+ – Input
Encoded peptide
Frequency
z-score

Fig. S1. Development of the AIMcap system to identify the targets of public TCRs. (**A**) Plot of % frequency (log scale) of specified TCRβ genes relative to all TCRβ genes sequenced in peripheral blood sample of donor. Each point represents an individual donor, and is colored according to CMV serological status (top) or according to HLA type (bottom). (**B**) Plot of $CD69^+$ versus $CD69^+IL\text{-}2^+$ Jurkat cells after co-culture of CMV-TCR #1-expressing T cells with aAPCs expressing HLA-B*07:02 alone or with the genome-wide CMV library, corresponding to Fig. 1D. *$P < 0.05$, ***$P < 0.0005$ compared to cells expressing HLA alone (Student's t-test). (**C**) Flow cytometric analysis of baseline CD69 expression versus IL-2 capture on Jurkat cells expressing EBV TCR #1, CMV-TCR #1, or CMV-TCR #2. Data are representative of three independent experiments. Bar plot graphing flow data is shown (right); *$P < 0.05$, **$P < 0.005$. (**D**) Flow cytometric analysis of CMV-TCR #1–expressing Jurkat cells showing APC anti-CD69 versus PE anti-IL2 (capture) after co-culture with aAPCs expressing HLA-B*07:02 without or with a dose curve of lentivirus that contains the cognate encoded peptide (TPRVTGGGAM). Data are representative of two independent experiments. (**E**) Plot of % $CD69^+IL\text{-}2^+$ Jurkat cells, corresponding to (D). *$P < 0.05$, **$P < 0.005$, and ***$P < 0.0005$ compared to cells expressing HLA alone (Student's t-test). (**F**) Plot of fold change of $CD69^+$ versus IL2 $capture^+$ Jurkat cells, corresponding to (D). The average baseline $CD69^+$ and IL2 $capture^+$ value was used as the denominator in calculating fold change. *$P < 0.05$, **$P < 0.005$, and ***$P < 0.0005$ of comparison at each fold change (Student's t-test). (**G**) Flow cytometric analysis of CMV-TCR #1– and CMV-TCR #2–expressing Jurkat cells showing APC anti-CD69 versus PE anti-IL2 (capture) after co-culture with aAPCs presenting the restricting HLA allele (HLA-B*07:02 and HLA-A*01:01, respectively) without or with either the CMV-TCR #1 or CMV-TCR #2 cognate peptide (TPRVTGGGAM and YSEHPTFTSQY, respectively). Data are representative of three independent experiments. (**H**–**J**) Flow cytometric analysis of aAPCs showing forward scatter (FSC) versus PE–streptavidin after proximity labeling. aAPCs expressing HLA-B*07:02 and HLA-A*01:01, along with TPRVTGGGAM or YSEHPTFTSQY (the target and non-target encoded peptide) were co-cultured with CMV-TCR #1– and CMV-TCR #2–expressing Jurkat cells, and then anti-CD2– (H), anti-CD69– (I), or anti-IL2–mediated (J) proximity labeling was performed. Data are representative of three independent experiments. (**K**) Percent of total NGS reads in the sorted and unsorted populations are graphed from the experiment in Fig. 1K. (**L**–**M**) Similar to Fig. 1K, HLA-B*07:02–, HLA-A*01:01–, and CEF32 library–expressing aAPCs were co-cultured with Jurkat cells expressing CMV-TCR #1 (top) or CMV-TCR #2 (bottom). After co-culture, anti-IL-2– (L) or anti-CD2–mediated (M) proximity labeling was performed. Stained cells were sorted, and the epitope-encoding DNA was amplified and sequenced. Difference between sorted cells and unlabeled cells in percent total NGS reads for each encoded peptide are graphed, along with the frequency of z score measurements for each epitope. Data are representative of 1–2 independent experiments.

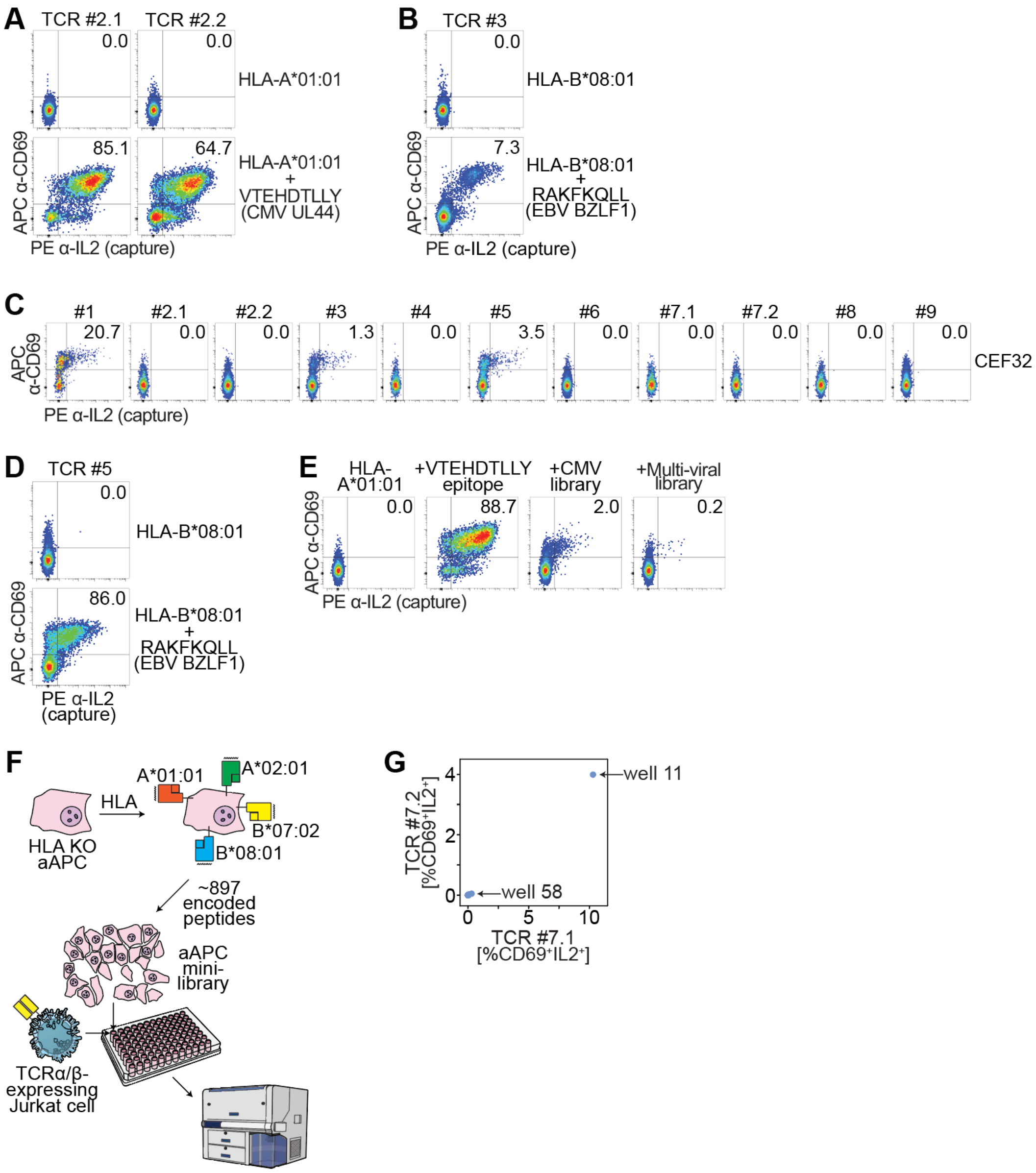

Fig. S2. Screening libraries of reference human viruses. (**A**) Flow cytometric analysis of TCR #2.1– or #2.2–expressing Jurkat cells showing APC anti-CD69 versus PE anti-IL-2 (capture) after co-culture with aAPCs expressing HLA-A*01:01 without or with the cognate CMV VTEHDTLLY epitope-encoding gene. Data are representative of three independent experiments. (**B**) Flow cytometric analysis of TCR #3-expressing Jurkat cells showing APC anti-CD69 versus PE anti-IL-2 (capture) after co-culture with aAPCs expressing HLA-B*08:01 without or with the cognate EBV RAKFKQLL epitope-encoding gene. Data are representative of three independent experiments. (**C**) Flow cytometric analysis of indicated TCR–expressing Jurkat cells showing APC anti-CD69 versus PE anti-IL-2 (capture) after co-culture with aAPCs expressing the restricting HLA allele with the CEF32 encoded peptide library. (**D**) Flow cytometric analysis of TCR #5-expressing Jurkat cells showing APC anti-CD69 versus PE anti-IL-2 (capture) after co-culture with aAPCs expressing HLA-B*08:01 without or with the cognate EBV RAKFKQLL epitope-encoding gene. Data are representative of three independent experiments. (**E**) Flow cytometric

analysis of TCR #2.1-expressing Jurkat cells showing APC anti-CD69 versus PE anti-IL-2 (capture) after co-culture with aAPCs expressing HLA-A*01:01 without or with the cognate CMV VTEHDTLLY epitope-encoding gene, the CMV exome library, or the multi-viral library. Data are representative of three independent experiments. (**F**) Schematic of the system to screen sub-libraries of the multi-viral library. HLA knockout aAPCs are transduced with HLA alleles. In each well of the 96-well plate, aAPCs are transduced with a sub-library of the multi-viral library, and then co-cultured with TCR-expressing Jurkat cells. After co-culture, Jurkat cells are strained for activation-induced markers and flow cytometry is performed. (**G**) Comparison of %$CD69^{+}IL\text{-}2^{+}$ Jurkat cells after co-culture of TCR #7.1– and #7.2-expressing Jurkat cells with aAPCs expressing each sub-library. Specific wells are labeled.

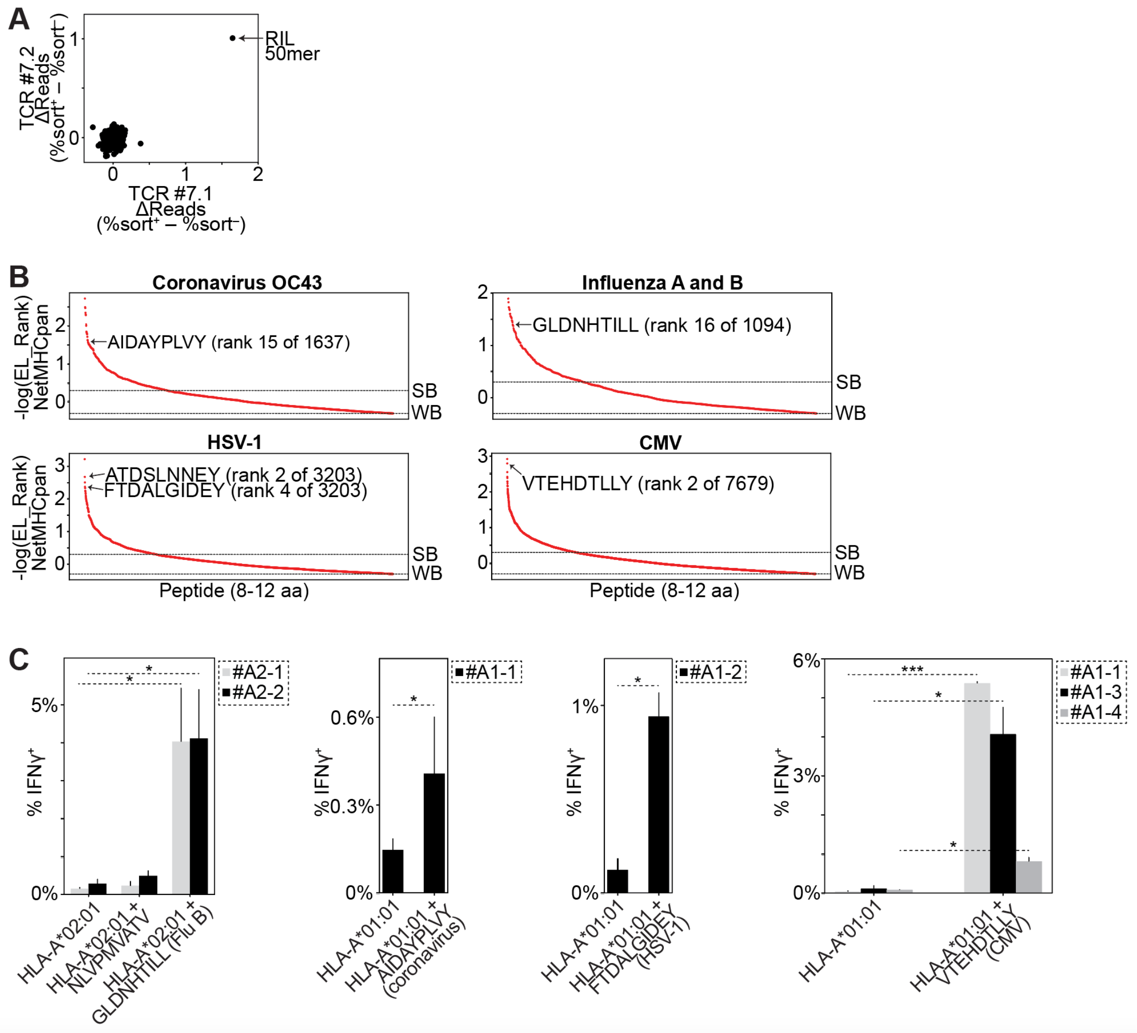

Fig. S3. Determination of the target viral epitope using proximity labeling. (**A**) Comparison between the enrichment of encoded peptides after proximity labeling using TCRs #7.1 and #7.2. Data corresponds to Fig. 3B. (**B)** %EL_Rank scores (−log) from NetMHCpan-4.1 for all 8–12 amino acid peptides in indicated viruses from the multi-viral library. Thresholds for strong binders (SB) and weak binders (WB) are marked. Specific epitopes are labeled. (**C**) Bar plots of IFNγ capture after co-culture of primary CD8$^+$ T cells from healthy donors with surface-anchored anti-IFNγ antibody-expressing aAPCs expressing indicated HLA alleles alone or with the indicated minimal encoded peptides. NLVPMVATV is a CMV peptide used as a control. Data are representative of two independent experiments. *$P < 0.05$, ***$P < 0.0005$ compared to aAPCs expressing HLA alone (Student's t-test).

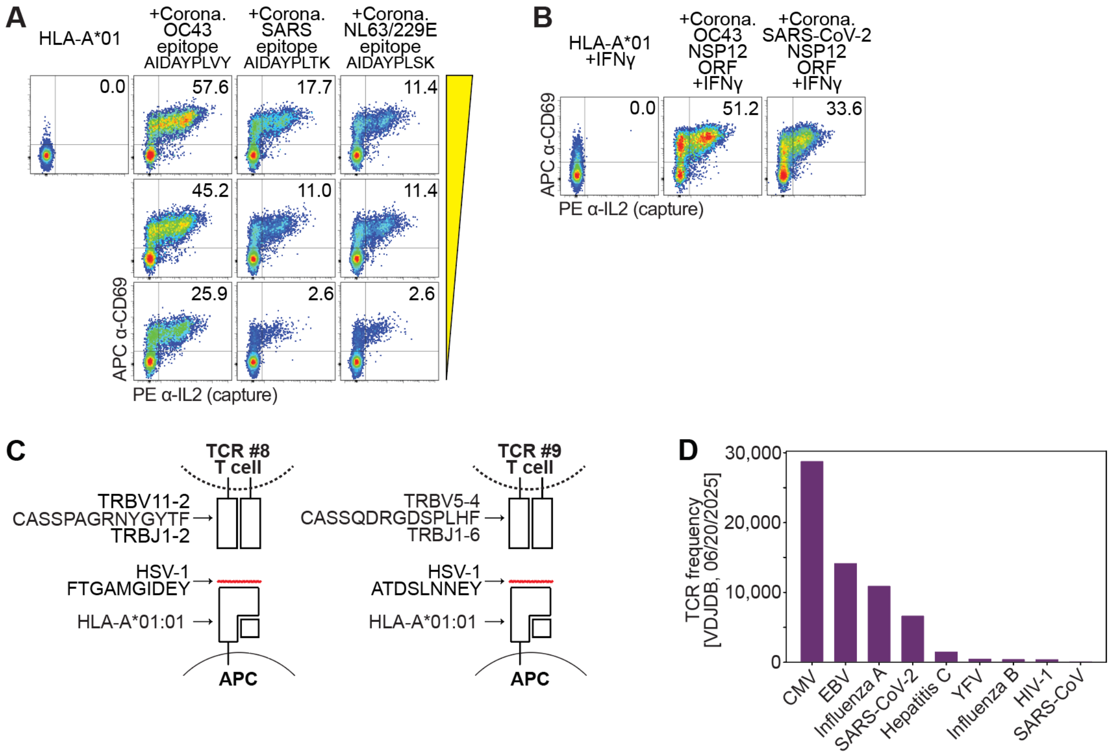

Fig. S4. Determination of viral cross-reactivity. (**A**) Flow cytometric analysis of TCR #7.1–expressing Jurkat cells showing APC anti-CD69 versus PE anti-IL-2 (capture) after co-culture with aAPCs expressing HLA-A*01:01 without or with a panel of encoded coronavirus peptides from Fig. 4A. A dose curve of lentivirus that contains each encoded peptide was used. (**B**) Flow cytometric analysis of TCR #7.1–expressing Jurkat cells showing APC anti-CD69 versus PE anti-IL-2 (capture) after co-culture with IFNy-treated aAPCs expressing HLA-A*01:01 without or with coronavirus NSP12 ORFs. Data are representative of three independent experiments. (**C**) Schematic representation of the complete TCR–peptide–HLA complexes for TCR #8 and #9. (**D**) Bar plot of the frequency of TCRβ sequences in VDJdb (taken from 06/20/2025) that are denoted as reactive to the indicated viruses; the top 9 most frequently denoted viruses in VDJDB are shown, which represent 99% of the viral-reactive TCRβ sequences.

Supplementary Tables.

Table S1 (separate file). Public TCR beta sequences.
Table S2 (separate file). Public single-cell TCR sequences.
Table S3 (separate file). Cloned public TCRs.
Table S4 (separate file). Multi-viral library.
Table S5 (separate file). Sequencing reads from AIMcap proximity labeling screens.
